# Global strategies and effectiveness for COVID-19 prevention through contact tracing, screening, quarantine, and isolation: a systematic review

**DOI:** 10.1186/s41182-020-00285-w

**Published:** 2020-11-23

**Authors:** Tadele Girum, Kifle Lentiro, Mulugeta Geremew, Biru Migora, Sisay Shewamare

**Affiliations:** 1 Department of Public Health, College of Medicine and Health Sciences, Wolkite University, Wolkite, Ethiopia; 2 Department of Statistics, College of natural and computational Sciences, Wolkite University, Wolkite, Ethiopia; 3 Department of Physics, College of natural and computational Sciences, Wolkite University, Wolkite, Ethiopia

**Keywords:** COVID-19, Quarantine, Contact tracing, Screening, Isolation

## Abstract

**Background:**

COVID-19 is an emerging disease caused by highly contagious virus called SARS-CoV-2. It caused an extensive health and economic burden around the globe. There is no proven effective treatment yet, except certain preventive mechanisms. Some studies assessing the effects of different preventive strategies have been published. However, there is no conclusive evidence. Therefore, this study aimed to review evidences related to COVID-19 prevention strategies achieved through contact tracing, screening, quarantine, and isolation to determine best practices.

**Methods:**

We conducted a systematic review in accordance with the PRISMA and Cochrane guidelines by searching articles from major medical databases such as PubMed/Medline, Global Health Database, Embase, CINAHL, Google Scholar, and clinical trial registries. Non-randomized and modeling articles published to date in areas of COVID prevention with contact tracing, screening, quarantine, and isolation were included. Two experts screened the articles and assessed risk of bias with ROBINS-I tool and certainty of evidence with GRADE approach. The findings were presented narratively and in tabular form.

**Results:**

We included 22 (9 observational and 13 modeling) studies. The studies consistently reported the benefit of quarantine, contact tracing, screening, and isolation in different settings. Model estimates indicated that quarantine of exposed people averted 44 to 81% of incident cases and 31 to 63% of deaths. Quarantine along with others can also halve the reproductive number and reduce the incidence, thus, shortening the epidemic period effectively. Early initiation of quarantine, operating large-scale screenings, strong contact tracing systems, and isolation of cases can effectively reduce the epidemic. However, adhering only to screening and isolation with lower coverage can miss more than 75% of asymptomatic cases; hence, it is not effective.

**Conclusion:**

Quarantine, contact tracing, screening, and isolation are effective measures of COVID-19 prevention, particularly when integrated together. In order to be more effective, quarantine should be implemented early and should cover a larger community.

## Introduction

Coronavirus disease 2019 (COVID-19) is an emerging infectious disease caused by severe acute respiratory syndrome coronavirus 2 (SARS-CoV-2). The novel coronavirus was first identified in December 2019 in Wuhan China, then spread globally within weeks and resulted in an ongoing pandemic [1–5]. Currently, coronavirus is affecting 213 countries and territories around the world. As of 27 May 2020, more than 5.7 million cases and 353,664 deaths were reported globally [2, 3]. Thirteen percent of the closed cohorts and 2–5% of the total cohort reportedly died [2–5]. The USA, Brazil, Russia, Spain, Italy, France, and the UK are the most affected countries [3–7].

The full spectrum of COVID-19 infection ranges from subclinical self-limiting respiratory tract illness to severe progressive pneumonia with multi-organ failure and death. As evidenced from studies and reports, more than 80% of cases remained asymptomatic and 15% of cases appeared as mild cases with common symptoms like fever, cough, fatigue, and loss of smell and taste [2–6]. Severe disease onset that needs intensive care might result in death due to massive alveolar damage and progressive respiratory failure [1, 4–8].

The virus transmits through direct and indirect contacts. Person-to-person transmissions primarily occur during close contact, droplets produced through coughing, sneezing, and talking. Indirect transmission occurs through touching contaminated surfaces or objects and then touching the face. It is more contagious during the first few days after the onset of symptoms, but asymptomatic cases can also spread the disease [5–8].

Recommended prevention measures was designed based on overcoming the mode of transmissions including frequent hand washing, maintaining physical distance, quarantine, covering the mouth and nose during coughs, and avoiding contamination of face with unwashed hands. In addition, use of mask is recommended particularly for suspected individuals and their caregivers. There is limited evidence against the community wide use of masks in healthy individuals. However, most of these preventive measures are recommended and were not researched well [4–8].

To the extent of our search, there is no systematic review on the preventive aspects and effectiveness of COVID-19 infection through contact tracing, screening, quarantine, and isolation. The findings were inconclusive; in some studies, certain preventive mechanisms were shown to have minimal effects, while in others different preventive mechanisms have better effect than expected. On the other hand, some studies have reported that integration of interventions is more effective than specific interventions [2, 6, 8].

Therefore, we aimed to conduct a comprehensive systematic review through reviewing globally published studies on the strategies and effectiveness of different preventive mechanisms (contact tracing, screening, quarantine, and isolation) developed to prevent and control COVID-19. This synthesized measure will be important to bring conclusive evidence, so that policy makers and other stakeholders could have clear evidence to rely on during decision making.

## Objectives

To support the existing local and national COVID-19 prevention program with tangible evidence, we conducted a systematic review on global strategies for COVID-19 prevention through contact tracing, screening, quarantine, and isolation. We aimed to answer issues related to alternative strategic implementation and effectiveness in the prevention of the disease or death. The following key questions were considered:

1. Is contact tracing, screening, quarantine, and isolation effective to control the COVID-19 outbreak?
2. Is there difference in the effectiveness of contact tracing, screening, quarantine, and isolation in different settings?
3. How and when these strategies should be applied to control the COVID-19 outbreak?

## Methods

We conducted the review in accordance with the PRISMA (Preferred Reporting Items for Systematic Reviews and Meta-Analyses) guidance for reporting of systematic reviews and meta-analyses [9] and the Cochrane Handbook of Systematic Review [10] through systematic literature search of articles published to date (June 02/2020) containing information on COVID-19 prevention by contact tracing, screening, quarantine, and isolation. First, a working protocol was developed (but unpublished) and followed in the process.

### Eligibility (inclusion and exclusion) criteria for the review

Based on the relevance of the reported evidence for decision making at local, national, and international levels, the papers were selected and prioritized for the review. The relevant outcomes observed in the review were reduction in incidence, transmission, adverse outcome, and cost-effectiveness of COVID-19 prevention through contact tracing, screening, quarantine, and isolation.

#### Types of studies

Due to the infancy of the epidemic, lack of researches, and ethical concerns, randomized controlled trials were not included. Therefore, we considered non-randomized observational studies and modeling (mathematical and/or epidemiological) studies to supplement the existing evidences.

We included cohort studies, case-control studies, time series, case series, and mathematical modeling studies conducted anywhere, in any area, and in any setting reported in the English language. Whereas, commentaries, letter to editor, case reports, and governmental reports were excluded.

#### Types of participants

Depending on the type of the research, for each preventive methods, different participants were included. These includes individuals who have had contacts with confirmed or suspected case of COVID-19, or individuals who lived in areas with COVID-19 outbreak, or individuals considered to be at high risk for COVID-19/suspected cases or cases of COVID-19 infection. The number of participants varies according to the individual researches. Individuals who have confirmed other symptomatic respiratory diseases were excluded.

#### Types of interventions

We included different types of interventions applied specifically or in combination, either voluntary or mandatory and in different settings (facility or community). In comparative studies, the interventions were compared with the non-applied groups or other comparison groups. We excluded interventions other than the aforementioned strategies.

#### Types of outcome measures

To identify the extent to which these interventions were applied globally and to measure their effectiveness in COVID-19 prevention, we used the following outcome measures: incidence of COVID-19, onward transmission, mortality or other adverse outcomes, and cost-effectiveness. We did not address secondary outcomes such as psychological impacts, economic impacts, and social impacts.

### Literature search strategy

A systematic literature search of articles was done by information system professionals and the researchers. Articles published between January 1, 2020, and June 2, 2020, containing information on different prevention strategies such as contact tracing, screening, quarantine, and isolation, and studies assessing their effectiveness were retained for the review. Electronic bibliographic databases and libraries such as PubMed/Medline, Global Health Database, Embase, CINAHL (Cumulative Index to Nursing and Allied Health Literature; Ebsco), the Cochrane Library, and African Index Medicus were used.

In addition, we searched gray literatures, pre-prints, and resource centers of *The Lancet*, *JAMA*, and *N Engl J Med*. Lastly, we screened the reference lists of systematic reviews for additional source. Combination of the following search terms were used with (AND, OR, NOT) Boolean (Search) Operators.

1. Corona virus
2. Coronavirus Infections
3. SARS COv2
4. COVID-19
5. Novel corona
6. Prevention/control
7. Contact tracing
8. Screening
9. Quarantine
10. Isolation
11. 1 or 2 or 3 or 4 or 5 and 6 and 7 or 8 or 9 or 10

### Data collection and analysis

#### Study selection process

The team screened all the titles and abstracts based on predefined eligibility criteria. Two authors independently screened the titles and abstracts and reached consensus by discussion or by involving a third author. After that, the review author team retrieved the full texts of all included abstracts. Two review authors screened all the full-text publications independently, and disagreements were resolved with consensus or by a third person involvement.

#### Data extraction and management

Titles and abstracts found through primary electronic search were thoroughly assessed for the possibility of reporting the intended outcome and filtered for potential eligibility. One of the review authors who have experience extracted data from the included studies into standardized tables, and the second author checked completeness. From each eligible research, the following information was extracted based on the preformed format: author information, title, study participants, study design, study setting, type of intervention, length of intervention, year of publication, study duration, eligibility criteria, rate, and effect of intervention measures. For modeling studies, the data extraction items also included the type of model and the data source.

#### Assessment of risk of bias in included studies

Risk of bias was assessed through evaluating reliability and validity of data in included studies based on the Risk-Of-Bias In Non-randomized Studies - of Interventions (ROBINS-I) tool [11]. The first author rated the risk of bias, the second author checked the ratings, and the third author was involved in the disagreements. For each studies, the study design, participants, outcome, and presence of bias were assessed based on the eligibility criteria and quality assessment check list. Moreover, all studies with the same participants and outcome were measured using the same standard.

On the other hand, modeling studies were assessed by the International Society for Pharmaco-economics and Outcomes (ISPOR) and the Society for Medical Decision making (SMDM) for dynamic mathematical transmission model tools [12]. Modeling studies that fulfilled all the three criteria were rated as “no concerns to minor concerns, ” and if one or more categories were unclear, it is rated as “moderate concerns,” and if one or more categories were not fulfilled, we had it rated as “major concerns.”

#### Data synthesis and analysis

The qualitative data was systematically reviewed and presented in accordance with the Cochrane guide line. We synthesized results from quantitative measures narratively and reported in tabular form. Because of the heterogeneity of the primary studies, quantitative analyses (meta-analysis) were not conducted.

#### Assessment of the certainty of the evidence

By using the GRADE approach [13], we graded the certainty of evidence for the main outcomes, reported in standard terms using tables. One of the authors conducted the certainty assessment which consists of assessments of risk of bias, indirectness, inconsistency, imprecision, and publication bias, and then, classified to one of the four categories: a high certainty (estimated effect lies close to the true effect), a moderate certainty (estimated effect is probably close to the true effect), a low certainty (estimated effect might substantially differ), and very low certainty (estimated effect is probably markedly different) from the true effect.

## Results

### Studies included

**Fig. 1:**
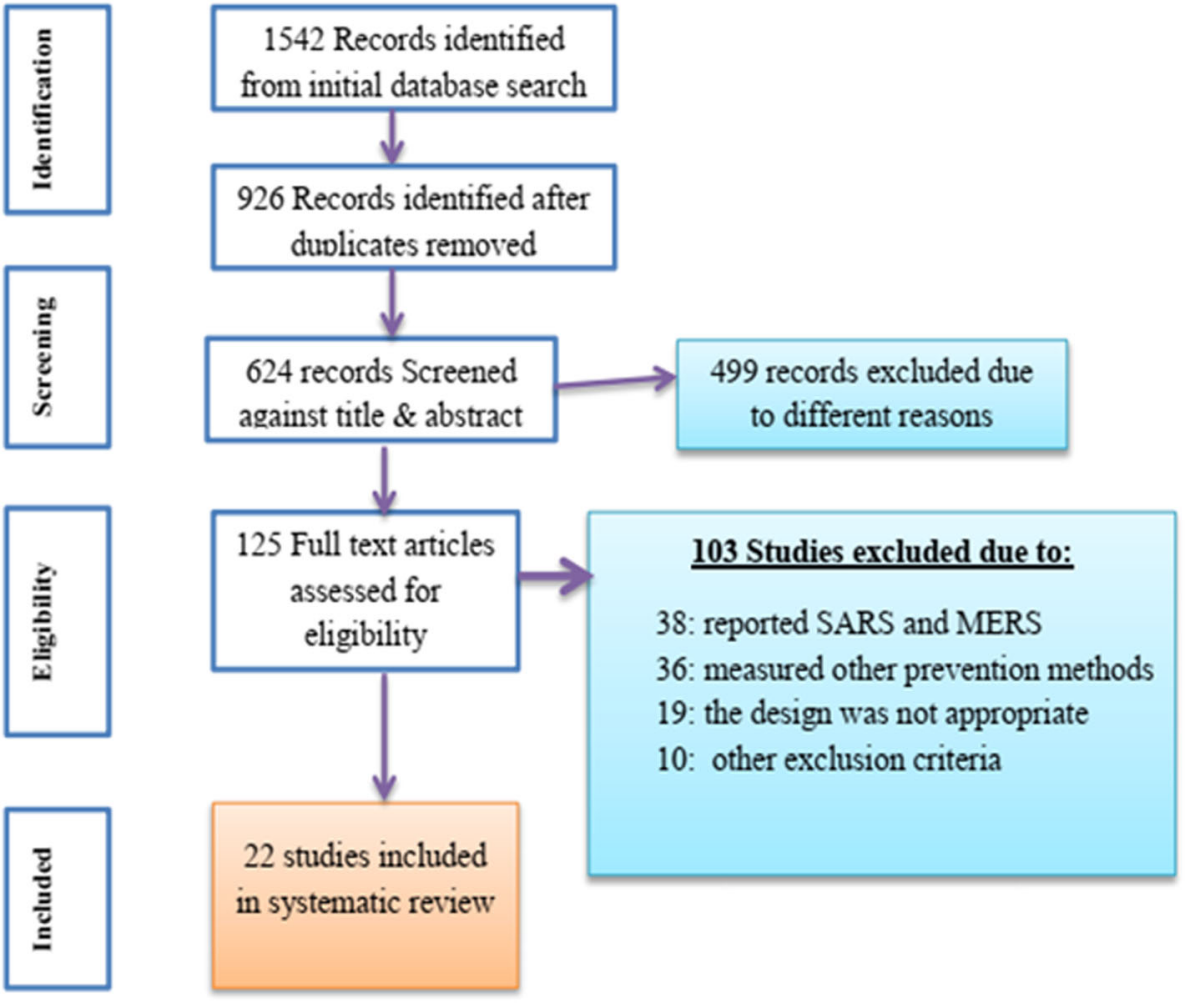
Flow chart for study search, selection, and screening for the review

The PRISMA flow diagram for the selected studies in the search process and the eligibility assessment are summarized in (Fig. 1). The initial electronic database search led to 1542 potentially relevant citations in the form of a title, abstract, bibliography, and full-text research. After removal of duplicates and initial screening, 125 articles were selected for further evaluation via full-text articles. Of these full-text articles, 103 articles were excluded due to the following reasons: 38 studies reported the prevention of SARS other than COVID-19; 36 have measured prevention measures other than contact tracing, screening, quarantine, and isolation; 19 had inappropriate study designs (commentaries, letters and case reports); and 10 were reviews or protocols. Thus, 22 studies [14–35] met the inclusion criteria and were included in the systematic review.

### Study characteristics

The 22 studies [14–35] that were retained for the final analysis were published in the period from January 15, 2020, to June 02, 2020, based on participant populations in the following countries: China (*n* = 10), UK (*n* = 4), USA (*n* = 2), Hong Kong (*n* = 2), and Netherlands, Japan, France, and Taiwan (*n* = 1 from each). The included studies comprised of 9 observational [14–22] and 13 modeling studies [23–35]. With duplicates (repeated count), 3 of the studies assessed the overall prevention strategies [21–23], 5 assessed the effect of contact tracing [14, 24, 25, 33, 35], 2 assessed screening strategies [17, 34], 12 assessed the effect of quarantine [15, 23–31], and 6 assessed the effect of isolation [17, 25, 26, 31, 33, 35]. The sample sizes in the studies varied from hundreds to millions. Four studies were investigated for effect at the health facility level, while the remaining 18 studies explored at the community or national level. Survey characteristics and summary results are described in Table 1.

**Table 1:** Characteristics of included studies and summary of result

S.N	Study characteristics and summary report	
1	Author/s and title [14]	Matt J Keeling, T Déirdre Hollingsworth, Jonathan M Read. The efficacy of contact tracing for the containment of the 2019 novel coronavirus (COVID-19).
	Population size (*N*)	5802
	Country	UK
	Setting	Community based
	Design	Cross-sectional study
	Objectives	To investigate the efficacy of contact tracing for the containment of Covid-19
	Study detail	Contact patterns was characterized using a postal and online cross-sectional survey
	Interventions	Contact tracing
	Results	Assuming that all the contact tracing can be performed rapidly, contact tracing to reduce the basic reproductive ratio from 3.11 to 0.21, enabling the outbreak to be contained. Each new case requires an average of 36 individuals to be traced, with 8.7% of cases having more than 100 close traceable contacts.
2	Author/s and title [15]	Zengyun Hu, Qianqian Cui, Junmei Han. Evaluation and prediction of the COVID-19 variations at different input population and quarantine strategies, a case study in Guangdong province, China
	Population size (*N*)	113460000
	Country	China
	Setting	Community based
	Design	Case study
	Objectives	To simulate and predict the disease variations of Guangdong province and to explore the impacts of the input population and quarantine strategies.
	Study detail	The impact of input population was evaluated with simulation
	Interventions	Quarantine
	Results	➢ The simulated peak value of the confirmed cases is 1002 at Feb 10, 2020 ➢ The disease will become extinction with peak value of 1397 at May 11, 2020. ➢ The increased numbers of the input population can mainly shorten the disease extinction days and the increased percentages of the exposed individuals
3	Author/s and title [16]	Mingwang S, Zhihang P, Yuming Gu, et al. Assessing the effects of metropolitan-wide quarantine on the spread of COVID-19 in public space and households
	Population size (*N*)	All population in Hubei province, China
	Country	China
	Setting	Community based
	Design	Retrospective
	Objectives	To evaluate the impact of the metropolitan-wide quarantine on the trend and transmission route of the SARS-CoV-2 epidemic
	Study detail	Data was collected on the number of cumulative confirmed cases
	Interventions	Quarantine
	Results	✓ In the presence of the quarantine, 100,610 infections, 68,975 confirmed cases and 3252 deaths would have occurred ✓ Quarantine would prevent 79.27% of deaths, 87.08% and 71.84% of infections in public space and households, respectively.
4	Author/s and title [17]	Jean C, Philippe C, et al. Testing the repatriated for SARS-Cov2: should laboratory-based quarantine replace traditional quarantine?
	Population size (*N*)	337
	Country	France
	Setting	Community based
	Design	Cross-sectional
	Objectives	To test all passengers for SARS-Cov2 twice in order to reduce anxiety among the population and decision makers
	Study detail	The presence of SARS-CoV-19 in asymptomatic carriers were investigated by testing all repatriated patients within the first 24 h of their arrival in France and at day 5, *n* = 337
	Interventions	Laboratory-based quarantine
	Results	✓ 337 passengers were tested at day 0 and day 5. ✓ Reducing the time scale to a matter of hours with molecular diagnosis is important
5	Author/s and title [18]	Hao-Yuan et al. Contact tracing assessment of COVID-19 transmission dynamics in Taiwan and risk at different exposure periods before and after symptom onset
	Population size (*N*)	100
	Country	Taiwan
	Setting	Community, health care setting
	Design	Prospective case-ascertained study
	Objectives	To delineate the transmission dynamics of COVID-19 and evaluate the transmission risk at different exposure window periods before and after symptom onset.
	Study detail	Prospective case-ascertained study that enrolled all the initial 100 confirmed cases
	Interventions	Contact tracing, all contacts were followed up until 14 days
	Results	✓ The overall secondary clinical attack rate was 0.7% (95% CI, 0.4–1.0%). ✓ The attack rate was higher among the 1818 contacts whose exposure to index cases started within 5 days of symptom onset. ✓ The 299 contacts with exclusive presymptomatic exposures were also at risk ✓ High transmissibility of COVID-19 before and immediately after symptom
6	Author/s and title [19]	Guan Wang, Wenhu Chen, Xian Jin, Yi-Peng Chen. Description of COVID-19 cases along with the measures taken on prevention and control in Zhejiang, China
	Population size (*N*)	Population in Hangzhou, Wenzhou, Ningbo, and Taizhou of Zhejiang Province
	Country	China
	Setting	Community based
	Design	Retrospective study
	Objectives	To perform a descriptive analysis of clinical characteristics and epidemiological factors of COVID-19 patients and summed up the steps for disease control and treatment in Zhejiang province.
	Study detail	Clinical characteristics were carried out on 889 confirmed cases
	Interventions	Screening, masks use, prohibiting public gathering, and suspending public transportation
	Results	➢ The factor of intimate contact with confirmed cases took up for 39%, 39%, 64%, and 44% in Hangzhou, Wenzhou, Ningbo, and Taizhou, respectively, which was the leading cause of COVID-19. ➢ Preventing contact with confirmed cases could largely avoid the disease to happen.
7	Author/s and title [20]	Siukan Law, et al. Severe acute respiratory syndrome (SARS) and coronavirus disease-2019 (COVID-19): from causes to preventions in Hong Kong
	Population size (*N*)	General population in Hong Kong
	Country	Hong Kong
	Setting	Community based
	Design	Retrospective
	Objectives	To discuss the current understanding of COVID-19 and compares with the outbreak of SARS-CoV-2 in 2003 of Hong Kong
	Study detail	A retrospective study was performed to summarize the current knowledge of COVID-19
	Interventions	Cases and contact handling and prevention activities on healthcare workers and community
	Results	Personal hygiene and protection are the most important for preventing the spread of COVID-19 such as wearing a mask and washing hands as well as reducing social contact including avoiding crowds and working at home.
8	Author/s and title [21]	Vincent C. et al. Escalating infection control response to the rapidly evolving epidemiology of the coronavirus disease 2019 (COVID-19) due to SARS-CoV-2 in Hong Kong
	Population size (*N*)	Population in Hong Kong
	Country	Hong Kong
	Setting	Health care setting
	Design	Case-control study, HCWs with unprotected exposure
	Objectives	To describe the infection control preparedness measures undertaken for coronavirus disease (COVID-19)
	Study detail	A bundled approach of active and enhanced laboratory surveillance, early airborne infection isolation, rapid molecular diagnostic testing, and contact tracing for healthcare workers (HCWs) with unprotected exposure in the hospitals was implemented
	Interventions	Isolation, rapid molecular diagnostic testing, and contact tracing
	Results	Vigilance in hand hygiene practice, wearing of surgical masks in the hospital, and appropriate use of PPE in patient care are the key infection control measures
9	Author/s and title [22]	Yansen Bai; et al. SARS-CoV-2 infection in health care workers: a retrospective analysis and model study
	Population size (*N*)	HCWs in the department of neurosurgery of union hospital of Wuhan, *N* = 171
	Country	China
	Setting	Health care setting
	Design	Single center of case-control series
	Objectives	➢ To investigate the risk factors to COVID-19.
	Study detail	A single-center study was carried out in the Department of Neurosurgery,
	Interventions	Quarantine and isolation
	Results	By reducing the average contact rate per HCW by a 1.35 factor and susceptibility by a 1.40 factor, we can avoid an outbreak of the basic case among HCWs.
10	Author/s and title [23]	Xiuli L. et al. Modelling the situation of COVID-19 and effects of different containment strategies in China with dynamic differential equations and parameters estimation
	Population size (*N*)	General population in china
	Country	China
	Setting	Community based
	Design	QSEIR modeling
	Objectives	To estimate the dynamic evolution mechanism of the epidemic in China, to find when the epidemic will end and how this result depends on different containment strategies.
	Study detail	A quantitative prediction of future epidemic developments based on different containment strategies with the QSEIR model has been made by setting January 23, 2020, as the beginning date of the simulation (5000)
	Interventions	Quarantine
	Results	Quarantine measures are the most effective containment strategy to control the epidemic.
11	Author/s and title [24]	Adam J. et al. Effectiveness of isolation, testing, contact tracing and physical distancing on reducing transmission of SARS-CoV-2 in different settings: modelling study
	Population size (*N*)	General population in UK
	Country	UK
	Setting	Community based
	Design	Mathematical modeling
	Objectives	To understand what combination of measures including novel digital tracing approaches and less intensive physical distancing may be required to reduce transmission.
	Study detail	Using a model of individual-level transmission stratified by setting (household, work, school, other) based on BBC Pandemic data from 40,162 UK participants
	Interventions	Isolation, testing, contact tracing, and physical distancing
	Results	➢ Combined isolation and tracing strategies would reduce transmission more than mass testing or self-isolation alone (50–60% compared to 2–30%).
12	Author/s and title [25]	Biao Tang, et al. Estimation of the transmission risk of the 2019-nCoV and its implication for public health interventions
	Population size (*N*)	Population in China
	Country	China
	Setting	Community based, health care
	Design	Mathematical Modeling, *R*_0_ = 6.47
	Objectives	To estimate the basic reproduction number by means of mathematical modeling
	Study detail	A deterministic compartmental model was devised based on the clinical progression of the disease, epidemiological status of the individuals, and intervention measures.
	Interventions	Contact tracing, quarantine and isolation
	Results	➢ The estimations based on likelihood and model analysis show that the control reproduction number may be as high as 6.47 (95% CI, 5.71–7.23). ➢ Interventions, such as intensive contact tracing followed by quarantine and isolation, can effectively reduce the control reproduction number ➢ With travel restriction, the number of infected individuals in seven days will decrease by 91.14% in Beijing, compared with the scenario of no travel restriction.
13	Author/s and title [26]	Rocklöv J, et al. COVID-19 outbreak on the Diamond Princess cruise ship: estimating the epidemic potential and effectiveness of public health countermeasures
	Population size (*N*)	Population in cruise ship
	Country	Japan
	Setting	Community based
	Design	SEIR modeling
	Objectives	To study the empirical data of COVID-19 confirmed infections on the Cruise ship Diamond Princess, to estimate the *R*_0_.
	Study detail	SEIR modeling was used on data confirmed cases on the cruise ship
	Interventions	Isolation, quarantine, and removal interventions
	Results	➢ Based on the modeled initial of 14.8, without any interventions within period of 21 January to 19 February, 2020, out of the 3700 (79%) would have been infected. ➢ The *R*_0_ was 14.8 initially and then declined to a stable 1.78 after the quarantine, and removal interventions were initiated. ➢ Isolation and quarantine therefore prevented 2307 cases
14	Author/s and title [27]	Zhao S, Chen H. Modeling the epidemic dynamics and control of COVID-19 outbreak in China
	Population size (*N*)	Population in china (excluding Hubei)
	Country	China
	Setting	Community based
	Design	Mathematical modeling, SUQC
	Objectives	To characterize the dynamics of COVID-19
	Study detail	SUQC model is applied to the daily released data
	Interventions	Quarantine
	Results	➢ The confirmation rate of Wuhan is 0.0643, substantially lower than that of Hubei excluding Wuhan (0.1914) and that of China excluding Hubei (0.2189), but it jumps to 0.3229 after February 12 when clinical evidence was adopted ➢ The number of unquarantined infected cases in Wuhan on February 12, 2020, is estimated to be 3509 and declines to 334 on February 21, 2020.
15	Author/s and title [28]	Neil M et al. Impact of non-pharmaceutical interventions (NPIs) to reduce COVID-19 mortality and healthcare demand
	Population size (*N*)	Population in the UK and USA
	Country	UK and USA
	Setting	Community based
	Design	Mathematical modeling study
	Objectives	To assess the potential role of a number of public health measures—so-called non-pharmaceutical interventions (NPIs)
	Study detail	The effect of non-pharmacological measures were measured
	Interventions	Non-pharmaceutical interventions
	Results	➢ To reduce *R*_0_ to close to 1 or below, a combination of case isolation, social distancing, quarantine, or school and university closure are required ➢ Optimal mitigation policies reduce healthcare demand by 2/3 and deaths by half.
16	Author/s and title [29]	Zifeng Yang, et al. Modified SEIR and AI prediction of the epidemics trend of COVID-19 in China under public health interventions
	Population size (*N*)	Population in china
	Country	China
	Setting	Community based
	Design	Mathematical Modeling, SEIR and an artificial intelligence (AI) approach
	Objectives	A modified susceptible-exposed-infected-removed (SEIR) epidemiological model was used that incorporates the domestic migration data before and after January 23 and the most recent COVID-19 epidemiological data to predict the epidemic progression.
	Study detail	SEIR model was used epidemiological data based on daily COVID-19 outbreak numbers reported by the National Health Commission of China
	Interventions	Quarantine, strict controls on travel and extensive monitoring of suspected cases
	Results	➢ A 5-day delay in implementation would have increased epidemic size three-fold. ➢ Where the interventions to be introduced 5 days earlier than they had been, the number of cases nationwide would have been 40,991 ➢ Lifting the Hubei quarantine would lead to a second epidemic peak in Hubei province in mid-March and extend the epidemic to late April
17	Author/s and title [30]	Peak, Corey M., et al. Modeling the comparative impact of individual quarantine vs. active monitoring of contacts for the mitigation of COVID-19 (2020)
	Population size (*N*)	2000
	Country	USA
	Setting	General population
	Design	Stochastic branching model
	Objectives	To estimate the comparative efficacy of these interventions to control COVID-19 using a stochastic branching model
	Study detail	A branching model was fitted for comparing two sets of reported parameters for the dynamics of the disease with a mean serial interval of 4.8 days and 7.5 days
	Interventions	Individual quarantine vs. active monitoring of contacts
	Results	➢ If social distancing reduces the reproductive number to 1.25 (e.g., 50% of person-to-person contact is removed in a setting where *R*_0_ = 2.5), active monitoring of 50% of contacts can result in overall outbreak control (i.e., *Re* < 1). ➢ Tracing 10%, 50%, or 90% of contacts on top of social distancing resulted in a median reduction in *R*_*e*_ of 3.2%, 15%, and 33%, respectively, for active monitoring and 5.8%, 32%, and 66%, for individual quarantine. ➢ Individual quarantine may contain an outbreak of COVID-19 with a short serial interval (4.8 days) only in settings with high intervention performance where at least three-quarters of infected contacts are individually quarantined.
18	Author/s and title [31]	Biao Tang et al. The effectiveness of quarantine and isolation determine the trend of the COVID-19 epidemics in the final phase of the current outbreak in China
	Population size (*N*)	General population in china
	Country	China
	Setting	Community based
	Design	dynamic model
	Objectives	To devise a dynamic model with suspected compartment incorporating prevention and control strategies to predict the trend of the COVID-19 epidemics based on multiple data sources and assess the efficacy of control strategies
	Study detail	data of laboratory-confirmed COVID-19 cases in China was obtained from the “National Health Commission” of the People’s Republic of China and the Hubei’s “Health Commission
	Interventions	Quarantine and isolation
	Results	✓ The trend of the epidemics mainly depends on quarantined and suspected cases. ✓ Most infected cases have been quarantined or put in suspected class, which has been ignored in existing models. ✓ The strong measures implemented have reduced the effective reproduction number. These interventions may take a longer time to be effective as the second and third generations of infected people are exposed in succession.
19	Author/s and title [32]	Can Hou, et al. The effectiveness of quarantine of Wuhan city against the corona virus disease 2019 (COVID-19): A well-mixed SEIR model analysis
	Population size (*N*)	11081000
	Country	China
	Setting	Community based
	Design	A well-mixed SEIR modeling
	Objectives	to explore the effectiveness of the quarantine of Wuhan city against the epidemic
	Study detail	The data of confirmed and suspected cases of COVID-19 acute respiratory disease reported by cities and provinces in mainland China were obtained
	Interventions	Quarantine
	Results	Reducing the contact rate of latent individuals after quarantine and isolation can effectively reduce the number of individuals infected with COVID-19 and delay the peak time.
20	Author/s and title [33]	Joel Hellewell, et al. Feasibility of controlling COVID-19 outbreaks by isolation of cases and contacts
	Population size (*N*)	100
	Country	UK
	Setting	Community based
	Design	Stochastic transmission modeling
	Objectives	To assess if isolation and contact tracing are able to control onwards transmission from imported cases of COVID-19
	Study detail	A mathematical model was employed to assess the feasibility of contact tracing and case isolation to control outbreaks of using simulated new outbreaks starting from 5, 20, or 40 introduced cases.
	Interventions	Contact tracing and case isolation
	Results	✓ When *R*_*0*_ was 2.5 or 3.5, the probability of controlling an outbreak decreased with the number of initial cases ✓ The majority of scenarios with an *R*_*0*_ of 1.5 were controllable with less than 50% of contacts successfully traced. ✓ To control the majority of outbreaks, for *R*_*0*_ of 2.5, more than 70% of contacts had to be traced, and for an *R*_*0*_ of 3.5, more than 90% of contacts had to be traced. ✓ The delay between symptom onset and isolation had the largest role in determining whether an outbreak was controllable when *R*_*0*_ was 1.5. ✓ For *R*_*0*_ values of 2.5 or 3.5, if there were 40 initial cases, contact tracing and isolation were only potentially feasible when less than 1% of transmission occurred before symptom onset. ✓ Contact tracing and isolation might not contain outbreaks of COVID-19 unless very high levels of contact tracing are achieved.
21	Author/s and title [34]	Katelyn Gostic, et al. Estimated effectiveness of symptom and risk screening to prevent the spread of COVID-19.
	Population size (*N*)	30
	Country	USA
	Setting	Community based
	Design	Mathematical modeling
	Objectives	To estimate the impact of different screening programs given current knowledge of key COVID-19 life history and epidemiological parameters
	Study detail	
	Interventions	Screening
	Results	✓ In a growing epidemic, even under the best-case assumptions, with just one infection in twenty being subclinical and all travelers passing through departure and arrival screening, the median fraction of infected travelers detected is only 0.30 ✓ In a stable epidemic, under the middle-case assumption that 25% of cases are subclinical, it is estimated that arrival screening alone would detect roughly one-third of infected travelers and that a combination of arrival and departure screening would detect nearly half of infected travelers ✓ Under best-case assumptions, screening will miss more than half of infected people
22	Author/s and title [35]	Mirjam E. et al. Isolation and contact tracing can tip the scale to containment of COVID-19 in populations with social distancing
	Population size (*N*)	100
	Country	Netherlands
	Setting	Community based
	Design	stochastic transmission model
	Objectives	To evaluate under which conditions containment could be achieved with combinations of social distancing, isolation and contact tracing
	Study detail	Stochastic transmission model-based analyses of the impact of isolation and contact tracing in a setting with various levels of social distancing measures, using varying levels of the effectiveness and timeliness of contact tracing was provided, *n* = 100.
	Interventions	Isolation and contact tracing in populations with social distancing
	Results	✓ If the proportion of asymptomatic infections is larger than 30%, contact tracing and isolation cannot achieve containment for an *R*_0_ of 2.5 ✓ To achieve containment by social distancing requires a reduction of numbers of non-household contacts by around 90%. ✓ Social distancing reduces non-household contacts only by 50%, tracing and isolation also of non-household contacts is needed for containment.

### Quality (risk of bias) assessment within included studies

Summaries of the risk of bias assessment of non-randomized studies and quality rating of the modeling studies are presented in Tables 2 and 3, respectively. Two studies [14, 19] have low bias due to confounding, eight studies have low bias in selection of participants into the study, and all studies have low bias in classification of interventions. The overall risk of bias is moderate for eight studies and serious for one study. On the other hand, we have no concern for nine modeling studies, and two studies have major concerns.

**Table 2:** Risk of bias assessment of observational studies based on ROBINS-I

Author and year	Bias due to confounding	Bias in selection of participants into the study	Bias in classification of interventions	Bias due to deviations from intended interventions	Bias due to missing data	Bias in measurement of outcomes	Bias in selection of the reported result	Overall risk of bias
Matt J 2020	Moderate	Low	Low	Low	Moderate	Moderate	Low	Moderate
Hu Z 2020 [15]	Moderate	Low	Low	Low	Moderate	Moderate	Low	Moderate
Shen 2020 [16]	Moderate	Low	Low	Low	Moderate	Moderate	Low	Moderate
Lagier 2020 [17]	Moderate	Low	Low	Low	Moderate	Low	Low	Serious
Cheng 2020 [18]	Moderate	Moderate	Low	Low	Moderate	Moderate	Low	Moderate
Wang 2020 [19]	Moderate	Low	Low	Low	Moderate	Moderate	Low	Moderate
Law 2020 [20]	Moderate	Low	Low	Low	Moderate	Moderate	Low	Moderate
Cheng 2020b [21]	Moderate	Low	Low	Low	Moderate	Moderate	Low	Moderate
Bai 2020 [22]	Moderate	Low	Low	Low	Moderate	Moderate	Low	Moderate

**Table 3:** Quality rating of the modeling studies based on three best practice recommendations from ISPOR

Author and year	Was the model a dynamic (transmission) model?	Did the authors conduct uncertainty analyses on key assumptions that may have had an impact of the conclusions?	Do the results provide estimates of the change in the burden of infection due to the intervention?	Quality
Xiuli 2020 [23]	Unclear	Yes	Unclear	Major concerns
Adam 2020 [24]	Yes	Yes	Yes	No concerns to minor concerns
Tang 2020 [25]	Yes	Yes	Yes	No concerns to minor concerns
Rocklöv 2020 [26]	Yes	Yes	Yes	No concerns to minor concerns
Zhao 2020 [27]	Unclear	Yes	Yes	Moderate concerns
Ferguson 2020	Yes	Yes	Yes	No concerns to minor concerns
Yang 2020 [29]	Yes	Yes	Yes	No concerns to minor concerns
Peak 2020 [30]	Yes	Yes	Yes	No concerns to minor concerns
Tang 2020 [31]	Yes	Yes	Yes	No concerns to minor concerns
Hou 2020 [32]	Yes	Yes	Yes	No concerns to minor concerns
Hellewell 2020 [33]	Yes	Yes	Yes	No concerns to minor concerns
Gostic 2020 [34]	Unclear	Yes	Yes	Moderate concerns
Mirjam 2020	Unclear	No	Unclear	Major concerns

### COVID-19 prevention strategies and effectiveness

The summary result is presented in Table 1. Among the nine observational studies, three of them assessed COVID-19 transmission with the existing prevention measures at a community level in Taiwan, China, and Hong Kong [18–20]. The other two studies assessed the effect of escalating prevention measures at health facilities in China and Hong Kong [21, 22], and three studies [15–17] assessed national- and metropolitan-based quarantine strategies and the effect of laboratory-based quarantine in the prevention of COVID-19. The last study evaluated the effect of community-based contact tracing in UK [14].

The three studies [18–20] that assessed the overall prevention strategies found out that integration of interventions need to be applied instead of adhering to a single intervention. Cheng [18] reported that isolating symptomatic patients alone may not be sufficient enough to contain the epidemic. Wang [19] and Law [20] also concluded that in intimate contacts the transmission is 40–60%. Preventing contact through different strategies and integration is very important.

Studies conducted on the effect of quarantine [15–17] found that it can have a massive preventive effect. One of the studies [15] that assessed the effect of quarantine in different populations and quarantine strategies found that it should be integrated with input population reduction (travel restriction), and the other study [16] that assessed the effects of metropolitan-wide quarantine on the Spread of COVID-19 in China found that quarantine would prevent 79.27% (75.10–83.45%) of deaths and 87.08% (84.68–89.49%) of infections. Also, the other researcher [17] evidenced that laboratory-based screenings accomplished within hours can enhance the efficiency of quarantine.

Two studies described infection control preparedness measures in health care settings of Hong Kong and China [21, 22]. One of these studies [21] reported that infection transmission is highly increased within a short period of time and multiplicity of infection prevention strategies were recommended for prevention in health care setups. The other study [22] also concluded that practicing working shift among professionals working in facilities can be used as strategy to prevent thetransmission of COVID infection.

A study conducted by Keeling et al. [14] assessed the efficacy of contact tracing for the containment of COVID-19 in the UK. The study evaluated the contact pattern of the community and concluded that rapid contact tracing to reduce the basic reproduction number (*R*_0_) from 3.11 to 0.21 enables the outbreak to be contained. Additionally, it was found that each new case requires an average of 36 individuals to be traced, with 8.7% of cases having more than 100 close traceable contacts.

In this review, we identified 13 modeling studies [23–35] that assessed the effectiveness of contact tracing, screening, quarantine, and isolation for prevention of COVID-19 in different settings and groups. The simulation was done in individual or group basis and with different assumptions. Most of these studies used a model parameter from Chinese reports.

Three of these researches [25–27] particularly emphasized on the way how the *R*_0_ can be reduced and the epidemic would be reduced. The simulation by Tang et al. [25] aimed to estimate the *R*_0_ of SARS-CoV-2 and infer the required effectiveness of isolation and quarantine to contain the outbreak. Their susceptible-exposed-infected-recovered (SEIR) model estimated *R*_0_ of 6.47 and generalized that 50% reduction of contact rate achieved by isolation and quarantine would decrease the confirmed cases by 44%; reducing contacts by 90% also can decrease the number of cases by 65%. The other researcher, Rocklov (27), by using data from the Diamond Princess Cruise ship, concluded that quarantine of passengers prevented 67% of cases and lowered the *R*_0_ from 14.8 to 1.78. Similarly, the reduction of *R*_0_ was achieved from quarantine [28].

In addition to these, five studies [24, 28, 30, 31, 35] which modeled the effectiveness of different public interventions consistently reported that integrated intervention is better than a single intervention. One of these research conducted in the UK [24] found that combined isolation and tracing strategies would reduce transmission more than mass testing or self-isolation alone (50–60% compared to 2–30%). The other study [28] also reported that with *R*_0_ of 2.4, a combination of case isolation and voluntary quarantine for 3 months could prevent 31% of deaths. The others also concluded that quarantine should be strict and integrated with contact tracing, screening, and other interventions [30, 31, 35].

Five modeling studies also assessed the effect of quarantine [23, 29, 32], contact tracing [33], and screening [34]. All of the studies [23, 29, 32] reported that quarantine has reduced the incidence of infection and shortened the duration of the epidemic. However, the effectiveness depends on the level of integration with other strategies. Similarly, model simulations that assessed the effect of contact tracing and screening reported that the strategies are effective. However, as the report of Hellewell [33] stated, contact tracing and isolation might not contain outbreaks of COVID-19 unless very high levels of contact tracing are achieved. Similarly, the other researcher [34] reported that in a stable epidemic, under the assumption that 25% of cases are subclinical, it is estimated that arrival screening alone would detect roughly one-third of infected travelers.

## Discussion

This study aimed to assess the effectiveness of contact tracing, screening, and quarantine and isolation to prevent COVID-19 infection by reviewing existing literatures. The review identified and systematically synthesized the findings of 22 studies (9 observational and 13 modeling studies) [14–35] to bring the best available evidence that policy makers and implementers can use in the process of infection prevention interventions.

The studies consistently reported the benefit of contact tracing, screening, quarantine, and isolation in the prevention of COVID-19. The effectiveness of quarantine in particular is very high. Compared to individuals without any intervention quarantined people exposed to a confirmed case highly averted infections and deaths [15, 23–31]. Also, the effectiveness of quarantine increases whenever it is implemented along with other prevention measures such as isolation, contact tracing, and travel ban [23–31]. Although, screening and contact tracing are very important to control the epidemic, early initiation, larger coverage, and integration with other programs are very important. Unless the level of contact tracing and screening is high, prevention through isolation only is very limited, as the screening programs misses 75% of cases [3, 24].

Quarantine measures applied alone or integrated with other measures were reported to be the most effective measures [25–31]. However, integration of quarantine with other public health measures increases the effectiveness and efficiency of the program [36]. Implementation of early quarantine measures makes the strategy a more cost effective one [28, 30]. Quarantine implemented as self-quarantine and group quarantine is effective at varying levels once effectively implemented [28, 32]. Total lockdown measures enhance the effectiveness of quarantine measures [15–19]. When laboratory tests are very fast, laboratory-based quarantine could be an effective in health care setups [17].

This evidence is in line with the finding of other reviews and modeling studies conducted to assess the effectiveness of these measures in the prevention of SARS, MERS, and COVID-19 [28, 35–37]. As reported before, combination of case isolation and voluntary quarantine for 3 months could prevent 31% of deaths compared to any single intervention. And adding social distancing on the previous interventions on people aged 70 years or older for 4 months increases the prevention proportion of deaths to 49%. It can also reduce the reproductive number by half; hence, it can tremendously reduce the incidence of infection, reduce the period of epidemic, and enhance effectiveness of control [28, 36].

Our findings also witnessed the effectiveness of contact tracing measures used for pandemic response efforts at multiple levels of health care systems. Isolation of suspected and confirmed patients and their contact is at the heart of the prevention strategy. However, for the contact tracing to be an effective measure, it has to be integrated with other measures such as quarantine and screening. Because larger shares of individuals are asymptomatic, contact tracing may be difficult in areas where contact recording is unachievable. According to world health organization, contact tracing is also one of the most essential and effective strategies to control the epidemic [14, 24, 25, 33, 35]. Other studies also evidenced the importance of contact tracing and isolation in different settings [36, 37].

The finding of our review revealed that screening and isolation are important measures of disease prevention [17, 25, 26, 31, 33, 35]. Most of the researches recommend high-risk group screening and contact cases screening in a resource-limited setting. However, these programs are effective when the screening capacity is higher and contact tracing is effective. Otherwise, screening and isolation programs miss more than half of cases and may not be implemented alone [25, 33, 35]. Also evidences from different countries indicated that screening and isolation measures are implemented along with other measures, yet their role in the prevention of the epidemic is high [2, 3, 8, 36, 37].

## Limitation

This review included a wide variety of study designs (observational and model studies); hence, it failed to include meta-analysis (statistical measures). Modeled studies also assume different scenarios, where it may not be true in the general cases. Also, the review has included only publications reported in the English language and open access resources.

## Conclusion and recommendation

Quarantine, contact tracing, screening, and isolation are effective measures of COVID-19 prevention, particularly whenever integrated together. In order to be more effective, quarantine should be implemented early and covers larger community. Controlling population travel will enhance the effectiveness of quarantine. Screening, contact tracing, and isolation are effective particularly in areas where contact tracing is easily attainable. Although screening is the effective measure recommended by the WHO, since the disease is asymptomatic, it may miss a larger share of the population. Therefore, this should be integrated with other preventive measures. In order to control the COVID-19 epidemic, the health care system should consider high level of contact tracing, early initiation of nationwide quarantine measures, increasing coverage of screening service, and preparing effective isolation centers.

## Data Availability

Please contact author for data requests.

## Abbreviations

COVID-19: Coronavirus disease 2019 MERS Middle East respiratory syndrome SARS Severe acute respiratory syndrome
PRISMA: Preferred Reporting Items for Systematic Reviews and Meta-Analyses *R*_0_ Basic reproduction number SEIR Susceptible-exposed-infected-recovered WHO World Health Organization
